# Roles of Glial Cells in Sculpting Inhibitory Synapses and Neural Circuits

**DOI:** 10.3389/fnmol.2017.00381

**Published:** 2017-11-13

**Authors:** Ji Won Um

**Affiliations:** Department of Brain and Cognitive Sciences, Daegu Gyeongbuk Institute of Science and Technology (DGIST), Daegu, South Korea

**Keywords:** astrocytes, glia, inhibitory synapse, neural circuits, neurons

## Abstract

Glial cells are essential for every aspect of normal neuronal development, synapse formation, and function in the central nervous system (CNS). Astrocytes secrete a variety of factors that regulate synaptic connectivity and circuit formation. Microglia also modulate synapse development through phagocytic activity. Most of the known actions of CNS glial cells are limited to roles at excitatory synapses. Nevertheless, studies have indicated that both astrocytes and microglia shape inhibitory synaptic connections through various mechanisms, including release of regulatory molecules, direct contact with synaptic terminals, and utilization of mediators in the extracellular matrix. This review summarizes recent investigations into the mechanisms underlying CNS glial cell-mediated inhibitory synapse development.

## Introduction

Synapses are the fundamental information-processing units underlying neuronal networks in the brain. It is across synapses that neurons receive excitatory synaptic inputs from neighboring glutamatergic neurons and inhibitory inputs from various γ-aminobutyric acid-expressing (GABAergic) interneurons. In particular, GABAergic interneurons play important roles in controlling the properties of pyramidal neurons, such as firing frequency, to shape the activity of neuronal networks, and contribute to the generation of cortical rhythms (Buzsáki and Draguhn, 2004; Bartos et al., 2007; Bonifazi et al., 2009; Jensen and Mazaheri, 2010; Kullmann, 2011). An imbalance in the ratio of excitatory to inhibitory (E/I) synaptic activity has emerged as a shared pathophysiological mechanism in several neuropsychiatric disorders, including autism spectrum disorder (ASD) and schizophrenia, and in neurological disorders such as epilepsy (Lee et al., 2017). Thus, investigations of the key molecular mechanisms underlying both excitatory and inhibitory synapse development collectively contribute to a comprehensive understanding of the pathophysiological mechanisms of brain disorders.

Over the past 20 years, numerous studies have shown that various types of glial cells actively and distinctively participate in the control of various neuronal processes in both the peripheral nervous system (PNS) and central nervous system (CNS; Pfrieger and Barres, 1996; Christopherson et al., 2005; Perea et al., 2009; Eroglu and Barres, 2010; Stipursky et al., 2011). Among these cell types, astrocytes have received the most attention because of their crucial roles in synapse formation, transmission and plasticity (Clarke and Barres, 2013; Baldwin and Eroglu, 2017). Astrocytes are not uniform throughout the CNS; rather, depending on the brain region, they exhibit differences in characteristics ranging from cell shape to protein composition (Chai et al., 2017). Thus, astrocytes may execute their differential functions in a brain region-specific manner. In this context, it has been reported that astrocyte-conditioned media (ACM) from different brain regions possess different excitatory synaptogenic properties, reflecting distinct expression profiles of astrocyte-derived synaptogenic molecules, such as glypicans, SPARC (secreted protein acidic and cysteine rich) and hevin (also known as SPARC-like 1 [SPARCL1]) in astrocytes from different brain regions (Buosi et al., 2017). Similarly, the inhibitory synaptogenic potential of astrocytes may also differ in distinct brain regions owing to the unique expression profiles of various glial genes, a potential that warrants further investigation.

Microglia also impact synaptic functions through release of specific molecules that influence the phagocytic activities involved in synapse elimination (Wu et al., 2015). Although the various mechanisms underlying glia-mediated excitatory synapse development have been well established (Nagler et al., 2001; Ullian et al., 2001, 2004; Risher et al., 2014), the roles of glial cells in inhibitory synapse development have only recently been investigated. In the present review, I focus on the actions of glial cells in orchestrating inhibitory synapse development and relevant neural circuits. Additionally, where possible I highlight the implications of these mechanisms for various brain disorders. The roles of glial cells in excitatory synapse development have been the subject of excellent recent reviews (Perea et al., 2009; Chung et al., 2015) and will not be addressed here in detail.

The main role of oligodendrocytes is to generate myelin sheaths around axons (Barres, 2008), but a few studies have shown that a new class of glial cells—proteoglycan NG2-positive oligodendrocyte precursor cells (OPCs)—receive the input from glutamatergic or GABAergic neurons (Bergles et al., 2000; Lin and Bergles, 2004; Lin et al., 2005). However, the precise function of these direct contacts remains to be investigated; thus, the related topic is not covered in the current review.

## Roles of Astrocytic Factors in Regulating Inhibitory Synapse Structure and Function

### Astrocyte-Secreted Factors

Results from various studies have indicated that astrocytes regulate synaptic transmission and plasticity, partly via the release of gliotransmitters, such as glutamate, D-serine or ATP, in response to activity-dependent calcium influx (Zhang et al., 2003; Fellin et al., 2004; Volterra and Meldolesi, 2005; Haydon and Carmignoto, 2006; Jourdain et al., 2007; Perea et al., 2009; Araque et al., 2014). For example, it has been shown that astrocyte-derived DISC1 (disrupted in schizophrenia-1) is involved in dendritic arborization and maturation of excitatory, but not inhibitory, synapses by modulating D-serine production in a hippocampal neuron-astrocyte coculture system (Xia et al., 2016). Astrocyte-derived ATP was recently shown to regulate the excitability of cholecystokinin (CCK)-positive interneurons through activation of P2Y1 purinergic receptors (Tan et al., 2017). In addition to the above-mentioned gliotransmitters, astrocytes release a number of substances including thrombospondin, hevin, SPARC, transforming growth factor-β1 (TGF-β1), glypican 4/6, semaphorin 3A, γ-protocadherin (γ-Pcdh), ephrin-A3, cholesterol and brain-derived neurotrophic factor (BDNF), that are involved in directing the formation of synapses and ultimately building specific neural circuits (Baldwin and Eroglu, 2017).

**Figure 1:**
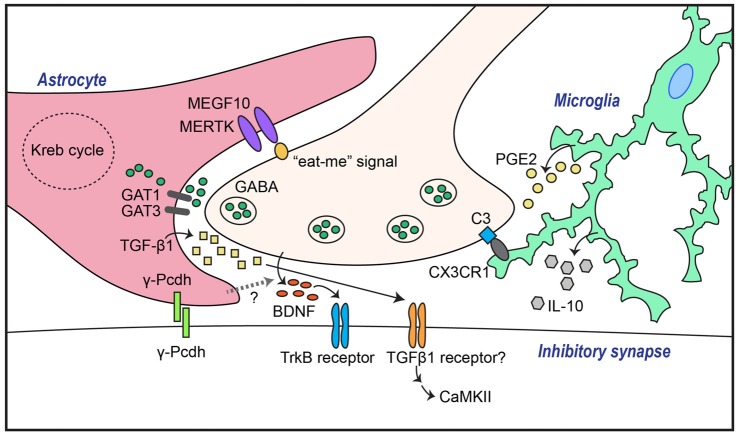
Astrocytes and microglia mediate both GABAergic synapse formation and elimination through a variety of molecular mechanisms. Transforming growth factor-β1 (TGF-β1) secreted from astrocytes induces inhibitory synapse formation through activation of neuronal calcium/calmodulin-dependent protein kinase II (CaMKII). In addition, GABAergic inhibitory synapse formation is regulated by astrocytic γ-Pcdh-mediated adhesion events, astrocytic GABA transporters (GATs), and/or unidentified factors that control neuronal brain-derived neurotrophic factor (BDNF)-TrkB signaling. Synapse elimination is mediated by astrocytic recognition of the so-called “eat-me” signal on neuronal membranes through pathways involving MEGF10 and MERTK, or by microglial recognition of complement C3 expression through complement receptor 3 (CR3), followed by phagocytosis.

In contrast, the impact of astrocytes on inhibitory synapse development has been largely unexplored. There are signaling pathways that link astrocytes with the GABA system, as suggested by earlier studies showing that astrocytes potentiate GABA-mediated currents in hippocampal cultured neurons (Liu et al., 1996, 1997). Moreover, astrocytes increase inhibitory synaptic transmission in hippocampal CA1 pyramidal neurons through astrocytic calcium signaling (Kang et al., 1998). Because the addition of ACM to neuronal cultures also induces GABAergic synapse-promoting effects similar to those observed in a neuron-astrocyte coculture system (Liu et al., 1996, 1997), it is possible that astrocytes secrete substances that are crucial for inhibitory synaptogenesis. In cultured hippocampal neurons, astrocyte-induced increases in the number of GABA_A_ receptor clusters were shown to be compromised by scavenging BDNF, indicating that signaling pathways involving BDNF and its receptor, tropomyosin receptor kinase B (TrkB), are required for astrocyte-mediated facilitation of inhibitory synapse development (Elmariah et al., 2005; Figure 1). Strikingly, astrocytic BDNF is not required for modulation of GABA_A_ receptor clustering, as evidenced by the fact that ACM from astrocytic-BDNF-deficient mice retains the ability to potentiate GABA_A_ receptor clustering (Elmariah et al., 2005). This suggests that unknown factors from astrocytes govern neuronal BDNF-TrkB signaling to promote inhibitory synapse development. In addition to modulating GABA_A_ receptor clustering, soluble astrocyte-derived factors selectively enhance axon length, branching, synapse number and function of GABAergic inhibitory neurons (Hughes et al., 2010). Intriguingly, thrombospondins, which positively regulate excitatory synapses, do not promote inhibitory synaptogenesis (Hughes et al., 2010).

Several astrocyte-secreted factors have recently been identified as inhibitory synapse regulators. For example, astrocyte-derived endozepines, endogenous ligands with benzodiazepine-like effects, potentiate synaptic inhibition in the thalamic reticular nucleus (Christian and Huguenard, 2013). In addition, TGF-β secreted by human and murine astrocytes induces inhibitory synapse formation in cortical cultured neurons (Diniz et al., 2014). In this latter study, disruption of calcium/calmodulin-dependent protein kinase II (CaMKII) function by either pharmacological inhibition or RNA interference (RNAi)-based knockdown abrogated ACM-triggered inhibitory synapse development, as assessed by clustering of inhibitory synaptic marker proteins (Diniz et al., 2014). Collectively, these results suggest that the TGF-β/CaMKII signaling pathway constitutes a key mechanism underlying astrocyte-mediated inhibitory synapse development (Figure 1), and that astrocytes regulate the synaptic E/I balance through a variety of molecular pathways.

### Extracellular Matrix Molecules

Astrocyte-derived ECM molecules are additional important factors that regulate key synaptic processes (Dityatev and Schachner, 2003; Christopherson et al., 2005; Faissner et al., 2010). Several studies have demonstrated that chondroitin sulfate proteoglycans (CSPGs) are involved in the regulation of synaptic plasticity (Pizzorusso et al., 2002; Frischknecht et al., 2009; Gogolla et al., 2009). Treatment with chondroitinase ABC (ChABC), an enzyme that eliminates CS chains, was shown to massively impair excitatory synaptic transmission in cultured hippocampal neurons (Pyka et al., 2011). However, inhibitory synaptic transmission was not affected (Pyka et al., 2011), indicating that the effects of CSPGs are restricted to excitatory synapses. Whether other ECMs are involved in specifically regulating inhibitory synapse development remains to be determined.

Perineuronal nets (PNNs)—specialized ECM structures surrounding neuronal soma and dendrites, particularly fast-spiking parvalbumin-positive (PV^+^) interneurons—inhibit synapse formation and reorganization (Sorg et al., 2016). Various proteoglycans, including neurocan, aggrecan, tenascins and hyaluronan, are concentrated in PNNs, and induction of their degradation by enzymatic treatment or genetic ablation leads to increased excitability of PV^+^ cells (Dityatev et al., 2007; Kim et al., 2016). PV^+^-interneurons are involved in the generation of synchronous γ-oscillations, which coordinate the activation of principal pyramidal neurons to maintain appropriate information processing and E/I balance, suggesting that components of PNNs involving PV^+^-interneurons may play an important role in fine-tuning the connectivity and/or activity of neural circuits. Disruption of this circuit formation manifest as pathophysiological correlates of discrete brain disorders, including epilepsy.

### Cell-Surface Proteins

Direct adhesion between neurons and astrocytes is also critical for synapse development. One factor that mediates astrocyte-neuron adhesion is γ-Pcdh, which promotes synaptogenesis through homophilic interactions (Frank and Kemler, 2002). Astrocytic γ-Pcdh promotes both excitatory and inhibitory synapse development, as revealed by genetic ablation of γ-Pcdh in either neurons or astrocytes (Garrett and Weiner, 2009; Figure 1). More than 20 adhesion proteins identified to date in pre- and postsynaptic membranes have been shown to organize various aspects of neuronal development processes (Um and Ko, 2013). However, whether these proteins are exclusively expressed in either neurons or glial cells, or both, has not been systematically investigated. Thus, it is conceivable that additional, as yet undiscovered, membrane proteins are involved in astrocyte-neuron adhesion processes.

Synapse elimination is crucial for normal synapse development across the CNS and PNS (Eroglu and Barres, 2010; Neniskyte and Gross, 2017). Excess synapses that form initially are removed during brain development to enable functional neural circuit formation. Recent studies have shown that astrocytes are involved in eliminating both excess excitatory and inhibitory synapse structures, likely through interactions of astrocytic multiple epidermal growth factor-like domains 10 (MEGF10) and MER proto-oncogene, tyrosine kinase (MERK) with unidentified neuronal membrane proteins (Chung et al., 2013, 2015). Phosphatidylserine, acting as an “eat-me” signal, drives remodeling of the synaptic architecture during brain development by binding to astrocytic MEGF10 and MERTK, which leads to phagocytosis (Chung et al., 2013; Figure 1). A recent study showed that astrocytes regulate synapse elimination through the release of ATP via a mechanism that is dependent on the type II inositol 1,4,5-triphosphate receptor (Yang et al., 2016). However, further studies are required to uncover the precise mechanisms underlying astrocyte-mediated synapse elimination. It further remains to be determined whether astrocytes are involved in eliminating both synapse types, and whether shared or distinct signaling pathways are involved in these processes. More importantly, how interactions of astrocytes, microglia, and neurons coordinate synapse elimination remains to be elucidated.

### Miscellaneous Factors

In addition to the mechanisms highlighted above, the modulation of GABAergic synapse development depends on a series of astrocytic metabolic pathways of the Krebs cycle (Kaczor et al., 2015; Kaczor and Mozrzymas, 2017). For example, inhibitory synapse number and transmission are increased and plasticity is enhanced in neurons cocultured with astrocytes compared to those cultured alone (Kaczor et al., 2015; Kaczor and Mozrzymas, 2017). These effects of astrocyte coculture disappear following treatment with a subset of selective Krebs cycle inhibitors, such as fluoroacetate, indicating the involvement of key astrocyte-expressed metabolic enzymes in GABAergic plasticity.

Because GABA in the extrasynaptic space shapes inhibitory synaptic transmission, it is conceivable that inhibitory synaptic transmission is regulated by the activity and/or level of GABA transporters (GATs). Four types of GATs (GAT1–4) have been identified in humans and rats. GAT1 and GAT3, in particular, are strongly expressed in astrocytes (Vitellaro-Zuccarello et al., 2003). Indeed, changes in astrocytic GAT1 or -3 expression level or activity alter inhibitory synaptic transmission in hippocampal interneurons (Beenhakker and Huguenard, 2010; Shigetomi et al., 2012; Kersanté et al., 2013; Muthukumar et al., 2014), suggesting that astrocytic GATs control the excitability of neurons in a neural network through regulation of extracellular GABA levels (Figure 1). Thus, astrocytic GATs may be considered potential therapeutic targets for neurological and psychiatric disorders.

## Roles of Astrocytes in Regulating the Formation of Inhibitory Inputs in Neural Circuits During Embryonic Development

Although the roles of glial cells in shaping neural circuits, particularly those that modulate GABAergic synaptic properties, remain largely unexplored, a few studies have implicated astrocytes in dictating the properties of discrete neural circuits. For example, in the auditory brainstem, inhibitory projections from the superior olivary nucleus (SON) to the nucleus laminaris (NL) are established during embryonic development (Burger et al., 2005). Treatment of organotypic slices from the avian auditory brainstem with ACM enhances the number of inhibitory synaptic inputs onto NL neurons, suggesting that soluble factors secreted by astrocytes promote inhibitory synaptogenesis during embryonic development (Korn et al., 2012; Cramer and Rubel, 2016). In the cerebellar cortex, Bergmann glial cells, a type of highly polarized astrocyte, guide stellate axons to form inhibitory synapses onto Purkinje neuronal dendrites during postnatal development (Ango et al., 2008), underscoring the importance of glial cells in shaping the cerebellar circuitry. Genetic deletion of specific developmental populations of astrocytes in the spinal cord was shown to increase inhibitory synapse numbers, but decrease excitatory synapse numbers (Tsai et al., 2012). These results indicate that astrocytes are crucial for maintaining the appropriate E/I ratio at synapses and neural circuits in the spinal cord. In the microcircuit connecting the thalamic reticular nucleus and ventrobasal nucleus, astrocytes regulate synaptic inhibition through endozepines and GATs (Khakh and Sofroniew, 2015). In addition, in the visual cortex, activation of astrocytes enhances the spontaneous firing rate of PV+ interneurons, contributing to shaping diverse sensory information-processing events in the primary visual cortical network (Perea et al., 2014; Ben Haim and Rowitch, 2017).

## Roles of Microglia in Inhibitory Synapse Formation and Elimination

Microglia are the resident macrophages in the CNS. In line with their immune cell identity, microglia have been traditionally investigated as mediators of inflammatory responses and phagocytosis of pathogens and cell debris under pathological conditions (Shemer et al., 2015). However, roles of microglia under normal conditions have recently begun to emerge. During postnatal development, microglia contribute to the reconstruction of neuronal circuits through phagocytosis of excess neuronal synapses and newborn neurons (Stevens et al., 2007; Paolicelli et al., 2011; Tremblay et al., 2011). A variety of molecules are responsible for phagocytosis-mediated synaptic pruning. These include CX3C chemokine receptor 1 (CX3XR1), a receptor of the neuronal chemokine fractalkine, CX3XL1, that is expressed exclusively in microglia (Paolicelli et al., 2011), and complement receptor 3 (CR3), a receptor for complement component C3 located at neuronal synapses (Schafer et al., 2012; Figure 1). In addition to their phagocytic activity, microglia also influence synapse development through the release of various factors, such as BDNF (Parkhurst et al., 2013), interleukin (IL)-10 (Lim et al., 2013), ATP (Pascual et al., 2012) and tumor necrosis factor α (TNFα; Lewitus et al., 2016). In terms of microglial regulation of inhibitory synapses, microglial IL-10 was found to promote both excitatory and inhibitory synapse development in cultured hippocampal neurons (Lim et al., 2013; Figure 1). In addition, a recent study demonstrated that activated microglia displace inhibitory GABAergic presynaptic terminals in adult mice, resulting in increased synchronized neuronal activity (Chen et al., 2014). Increased neuronal activity causes an elevation in intracellular calcium levels, leading to activation of CaMKII and increased expression of anti-apoptotic proteins (Chen et al., 2014). These data suggest a novel role of activated microglia in protecting the adult brain in addition to their phagocytic role (Chen et al., 2014). Further studies are required to establish the molecular factors involved in evoking protective microglia in brain disease states. In addition to GABAergic synapses, glycinergic inhibitory synapses are also regulated by microglia (Cantaut-Belarif et al., 2017). Stimulated microglia acutely regulate glycinergic synapse development in the spinal cord by modulating the activity of microglial prostaglandin E2 (PGE2; Cantaut-Belarif et al., 2017; Figure 1).

## Disease Relevance

Synaptic dysfunction has been considered a hallmark of various neurological diseases, including Alzheimer disease (AD), ASD and schizophrenia (Penzes et al., 2011; Zoghbi and Bear, 2012; Li et al., 2017). Both astrocytes and microglia influence synapse formation and elimination; thus, it is likely that impaired glial function contributes to the onset and progression of neurological disorders. Specifically, dysregulation of glial functions that disrupts the E/I balance at synapses and circuits may lead to disease states. Recently, a rare variant of a microglial gene encoding triggering receptor expressed on myeloid cell 2 (TREM2) has been identified as a risk factor for AD (Jonsson et al., 2013). In addition, microglial activation has been demonstrated in the brains of individuals with ASD or schizophrenia (van Berckel et al., 2008; Voineagu et al., 2011). However, it is not clear how microglial activation is related to synaptic deficits in these diseases. Also, because most published reports have focused on the roles of various glial cells in regulating excitatory synapse formation, function or elimination, the issue of whether glial cells also play critical roles in controlling inhibitory synapse development and function remains to be investigated.

Reactive astrocytes have been associated with many neurological diseases, including epilepsy, AD and stroke (Seifert et al., 2006). Mounting evidence has demonstrated multifaceted functions of reactive astrocytes in disease states, but little is known about the roles of these astrocytes from an inhibitory synapse or circuit perspective. One study showed that, in the astrocytotic region, neurons exhibit reduced inhibitory, but not excitatory, synaptic transmission through actions of the astrocytic glutamate-glutamine cycle, which triggered hyperexcitability in hippocampal circuits (Ortinski et al., 2010). Given the significance of GABAergic inhibition in neuronal circuits, these studies underscore the functional consequences of astrocytosis for neurological diseases as well as alterations of neuronal circuits, with attendant effects on cognition, learning and memory and epileptic seizures.

Extensive evidence has linked microglia to neuroinflammation, which in turn is associated with a variety of neurodegenerative diseases. However, impacts of microglia on the sculpting of synaptic connectivity have only recently been reported. Microglia in the healthy brain have been shown to function in the refinement of synapses in brain development, as described above. Disruption of microglial complement proteins or receptor proteins results in abnormal synaptic wiring (Paolicelli et al., 2011; Schafer et al., 2012), which may contribute to the synaptic abnormalities observed in several neurodevelopmental disorders.

## Concluding Remarks

In this review, I have highlighted recent literature reports that collectively reveal the various roles of astrocytes and microglia in regulating inhibitory synapse development and neural circuits. Considered in light of the essential role of GABAergic synapses in shaping network activity through filtering of incoming neural information and dictating the activity of principal neurons, the cellular and molecular mechanisms underlying inhibitory synapse structure, transmission, and plasticity mediated by various glial cell types should be comprehensible. Although recent technological developments have accelerated advances in our understanding of the roles of glial cells in various aspects of synapse development, only a few studies have provided mechanistic insights into the contributions of various glial cell types to the development of GABAergic synapses and relevant neural circuits. Investigations of unidentified astrocyte- and microglia-based mechanisms that direct the development of GABAergic synapses and neural circuits will not only enhance our understanding of synapse development in health, but also guide the development of novel therapeutic strategies against various brain disorders.

## Author Contributions

JWU wrote the manuscript.

## Conflict of Interest Statement

The author declares that the research was conducted in the absence of any commercial or financial relationships that could be construed as a potential conflict of interest.

## References

[B1] Ango F. Wu C. Van der Want J. J. Wu P. Schachner M. Huang Z. J. (2008). Bergmann glia and the recognition molecule CHL1 organize GABAergic axons and direct innervation of Purkinje cell dendrites. PLoS Biol. 6:e103. 10.1371/journal.pbio.0060103 18447583 PMC2689695

[B2] Araque A. Carmignoto G. Haydon P. G. Oliet S. H. Robitaille R. Volterra A. (2014). Gliotransmitters travel in time and space. Neuron 81, 728–739. 10.1016/j.neuron.2014.02.007 24559669 PMC4107238

[B3] Baldwin K. T. Eroglu C. (2017). Molecular mechanisms of astrocyte-induced synaptogenesis. Curr. Opin. Neurobiol. 45, 113–120. 10.1016/j.conb.2017.05.006 28570864 PMC5573249

[B4] Barres B. A. (2008). The mystery and magic of glia: a perspective on their roles in health and disease. Neuron 60, 430–440. 10.1016/j.neuron.2008.10.013 18995817

[B5] Bartos M. Vida I. Jonas P. (2007). Synaptic mechanisms of synchronized γ oscillations in inhibitory interneuron networks. Nat. Rev. Neurosci. 8, 45–56. 10.1038/nrn2044 17180162

[B6] Beenhakker M. P. Huguenard J. R. (2010). Astrocytes as gatekeepers of GABA_B_ receptor function. J. Neurosci. 30, 15262–15276. 10.1523/JNEUROSCI.3243-10.2010 21068331 PMC3056552

[B31] Ben Haim L. Rowitch D. H. (2017). Functional diversity of astrocytes in neural circuit regulation. Nat. Rev. Neurosci. 18, 31–41. 10.1038/nrn.2016.159 27904142

[B7] Bergles D. E. Roberts J. D. Somogyi P. Jahr C. E. (2000). Glutamatergic synapses on oligodendrocyte precursor cells in the hippocampus. Nature 405, 187–191. 10.1038/35012083 10821275

[B8] Bonifazi P. Goldin M. Picardo M. A. Jorquera I. Cattani A. Bianconi G.. (2009). GABAergic hub neurons orchestrate synchrony in developing hippocampal networks. Science 326, 1419–1424. 10.1126/science.1175509 19965761

[B9] Buosi A. S. Matias I. Araujo A. P. Batista C. Gomes F. C. (2017). Heterogeneity in synaptogenic profile of astrocytes from different brain regions. Mol. Neurobiol. [Epub ahead of print]. 10.1007/s12035-016-0343-z 28050794

[B10] Burger R. M. Cramer K. S. Pfeiffer J. D. Rubel E. W. (2005). Avian superior olivary nucleus provides divergent inhibitory input to parallel auditory pathways. J. Comp. Neurol. 481, 6–18. 10.1002/cne.20334 15558730

[B11] Buzsáki G. Draguhn A. (2004). Neuronal oscillations in cortical networks. Science 304, 1926–1929. 10.1126/science.1099745 15218136

[B12] Cantaut-Belarif Y. Antri M. Pizzarelli R. Colasse S. Vaccari I. Soares S.. (2017). Microglia control the glycinergic but not the GABAergic synapses via prostaglandin E2 in the spinal cord. J. Cell Biol. 216, 2979–2989. 10.1083/jcb.201607048 28716844 PMC5584146

[B13] Chai H. Diaz-Castro B. Shigetomi E. Monte E. Octeau J. C. Yu X.. (2017). Neural circuit-specialized astrocytes: transcriptomic, proteomic, morphological, and functional evidence. Neuron 95, 531.e9–549.e9. 10.1016/j.neuron.2017.06.029 28712653 PMC5811312

[B14] Chen Z. Jalabi W. Hu W. Park H. J. Gale J. T. Kidd G. J.. (2014). Microglial displacement of inhibitory synapses provides neuroprotection in the adult brain. Nat. Commun. 5:4486. 10.1038/ncomms5486 25047355 PMC4109015

[B89] Christian C. A. Huguenard J. R. (2013). Astrocytes potentiate GABAergic transmission in the thalamic reticular nucleus via endozepine signaling. Proc. Natl. Acad. Sci. U S A 110, 20278–20283. 10.1073/pnas.1318031110 24262146 PMC3864346

[B15] Christopherson K. S. Ullian E. M. Stokes C. C. A. Mullowney C. E. Hell J. W. Agah A.. (2005). Thrombospondins are astrocyte-secreted proteins that promote CNS synaptogenesis. Cell 120, 421–433. 10.1016/j.cell.2004.12.020 15707899

[B16] Chung W. S. Allen N. J. Eroglu C. (2015). Astrocytes control synapse formation, function and elimination. Cold Spring Harb. Perspect. Biol. 7:a020370. 10.1101/cshperspect.a020370 25663667 PMC4527946

[B17] Chung W.-S. Clarke L. E. Wang G. X. Stafford B. K. Sher A. Chakraborty C.. (2013). Astrocytes mediate synapse elimination through MEGF10 and MERTK pathways. Nature 504, 394–400. 10.1038/nature12776 24270812 PMC3969024

[B18] Clarke L. E. Barres B. A. (2013). Emerging roles of astrocytes in neural circuit development. Nat. Rev. Neurosci. 14, 311–321. 10.1038/nrn3484 23595014 PMC4431630

[B19] Cramer K. S. Rubel E. W. (2016). Glial cell contributions to auditory brainstem development. Front. Neural Circuits 10:83. 10.3389/fncir.2016.00083 27818624 PMC5073128

[B20] Diniz L. P. Tortelli V. Garcia M. N. Araújo A. P. Melo H. M. Silva G. S.. (2014). Astrocyte transforming growth factor β1 promotes inhibitory synapse formation via CaM kinase II signaling. Glia 62, 1917–1931. 10.1002/glia.22713 25042347

[B22] Dityatev A. Brückner G. Dityateva G. Grosche J. Kleene R. Schachner M. (2007). Activity-dependent formation and functions of chondroitin sulfate-rich extracellular matrix of perineuronal nets. Dev. Neurobiol. 67, 570–588. 10.1002/dneu.20361 17443809

[B21] Dityatev A. Schachner M. (2003). Extracellular matrix molecules and synaptic plasticity. Nat. Rev. Neurosci. 4, 456–468. 10.1038/nrn1115 12778118

[B23] Elmariah S. B. Oh E. J. Hughes E. G. Balice-Gordon R. J. (2005). Astrocytes regulate inhibitory synapse formation via Trk-mediated modulation of postsynaptic GABA_A_ receptors. J. Neurosci. 25, 3638–3650. 10.1523/JNEUROSCI.3980-04.2005 15814795 PMC6725365

[B24] Eroglu C. Barres B. A. (2010). Regulation of synaptic connectivity by glia. Nature 468, 223–231. 10.1038/nature09612 21068831 PMC4431554

[B25] Faissner A. Pyka M. Geissler M. Sobik T. Frischknecht R. Gundelfinger E. D.. (2010). Contributions of astrocytes to synapse formation and maturation—Potential functions of the perisynaptic extracellular matrix. Brain Res. Rev. 63, 26–38. 10.1016/j.brainresrev.2010.01.001 20096729

[B26] Fellin T. Pascual O. Gobbo S. Pozzan T. Haydon P. G. Carmignoto G. (2004). Neuronal synchrony mediated by astrocytic glutamate through activation of extrasynaptic NMDA receptors. Neuron 43, 729–743. 10.1016/j.neuron.2004.08.011 15339653

[B27] Frank M. Kemler R. (2002). Protocadherins. Curr. Opin. Cell Biol. 14, 557–562. 10.1016/S0955-0674(02)00365-4 12231349

[B28] Frischknecht R. Heine M. Perrais D. Seidenbecher C. I. Choquet D. Gundelfinger E. D. (2009). Brain extracellular matrix affects AMPA receptor lateral mobility and short-term synaptic plasticity. Nat. Neurosci. 12, 897–904. 10.1038/nn.2338 19483686

[B29] Garrett A. M. Weiner J. A. (2009). Control of CNS synapse development by γ-Protocadherin-mediated astrocyte-neuron contact. J. Neurosci. 29, 11723–11731. 10.1523/jneurosci.2818-09.2009 19776259 PMC2778296

[B30] Gogolla N. Caroni P. Lüthi A. Herry C. (2009). Perineuronal nets protect fear memories from erasure. Science 325, 1258–1261. 10.1126/science.1174146 19729657

[B32] Haydon P. G. Carmignoto G. (2006). Astrocyte control of synaptic transmission and neurovascular coupling. Physiol. Rev. 86, 1009–1031. 10.1152/physrev.00049.2005 16816144

[B33] Hughes E. G. Elmariah S. B. Balice-Gordon R. J. (2010). Astrocyte secreted proteins selectively increase hippocampal GABAergic axon length, branching, and synaptogenesis. Mol. Cell. Neurosci. 43, 136–145. 10.1016/j.mcn.2009.10.004 19850128 PMC2818511

[B34] Jensen O. Mazaheri A. (2010). Shaping functional architecture by oscillatory α activity: gating by inhibition. Front. Hum. Neurosci. 4:186. 10.3389/fnhum.2010.00186 21119777 PMC2990626

[B35] Jonsson T. Stefansson H. Steinberg S. Jonsdottir I. Jonsson P. V. Snaedal J.. (2013). Variant of TREM2 associated with the risk of Alzheimer’s disease. N Engl J. Med. 368, 107–116. 10.1056/NEJMoa1211103 23150908 PMC3677583

[B36] Jourdain P. Bergersen L. H. Bhaukaurally K. Bezzi P. Santello M. Domercq M.. (2007). Glutamate exocytosis from astrocytes controls synaptic strength. Nat. Neurosci. 10, 331–339. 10.1038/nn1849 17310248

[B38] Kaczor P. T. Mozrzymas J. W. (2017). Key metabolic enzymes underlying astrocytic upregulation of GABAergic plasticity. Front. Cell. Neurosci. 11:144. 10.3389/fncel.2017.00144 28559800 PMC5432623

[B37] Kaczor P. Rakus D. Mozrzymas J. W. (2015). Neuron-astrocyte interaction enhance GABAergic synaptic transmission in a manner dependent on key metabolic enzymes. Front. Cell. Neurosci. 9:120. 10.3389/fncel.2015.00120 25914620 PMC4391237

[B39] Kang J. Jiang L. Goldman S. A. Nedergaard M. (1998). Astrocyte-mediated potentiation of inhibitory synaptic transmission. Nat. Neurosci. 1, 683–692. 10.1038/3684 10196584

[B40] Kersanté F. Rowley S. C. S. Pavlov I. Gutièrrez-Mecinas M. Semyanov A. Reul J. M. H. M.. (2013). A functional role for both γ-aminobutyric acid (GABA) transporter-1 and GABA transporter-3 in the modulation of extracellular GABA and GABAergic tonic conductances in the rat hippocampus. J. Physiol. 591, 2429–2441. 10.1113/jphysiol.2012.246298 23381899 PMC3678035

[B41] Khakh B. S. Sofroniew M. V. (2015). Diversity of astrocyte functions and phenotypes in neural circuits. Nat. Neurosci. 18, 942–952. 10.1038/nn.4043 26108722 PMC5258184

[B42] Kim S. Y. Porter B. E. Friedman A. Kaufer D. (2016). A potential role for glia-derived extracellular matrix remodeling in postinjury epilepsy. J. Neurosci. Res. 94, 794–803. 10.1002/jnr.23758 27265805

[B43] Korn M. J. Koppel S. J. Li L. H. Mehta D. Mehta S. B. Seidl A. H.. (2012). Astrocyte-secreted factors modulate the developmental distribution of inhibitory synapses in nucleus laminaris of the avian auditory brainstem. J. Comp. Neurol. 520, 1262–1277. 10.1002/cne.22786 22020566 PMC3926803

[B44] Kullmann D. M. (2011). Interneuron networks in the hippocampus. Curr. Opin. Neurobiol. 21, 709–716. 10.1016/j.conb.2011.05.006 21636266

[B45] Lee E. Lee J. Kim E. (2017). Excitation/inhibition imbalance in animal models of autism spectrum disorders. Biol. Psychiatry 81, 838–847. 10.1016/j.biopsych.2016.05.011 27450033

[B46] Lewitus G. M. Konefal S. C. Greenhalgh A. D. Pribiag H. Augereau K. Stellwagen D. (2016). Microglial TNF-α suppresses cocaine-induced plasticity and behavioral sensitization. Neuron 90, 483–491. 10.1016/j.neuron.2016.03.030 27112496 PMC4860141

[B47] Li K. Wei Q. Liu F.-F. Hu F. Xie A.-J. Zhu L.-Q.. (2017). Synaptic dysfunction in Alzheimer’s disease: aβ, tau, and epigenetic alterations. Mol. Neurobiol. [Epub ahead of print]. 10.1007/s12035-017-0533-3 28456942

[B48] Lim S. H. Park E. You B. Jung Y. Park A. R. Park S. G.. (2013). Neuronal synapse formation induced by microglia and interleukin 10. PLoS One 8:e81218. 10.1371/journal.pone.0081218 24278397 PMC3838367

[B49] Lin S.-C. Bergles D. E. (2004). Synaptic signaling between GABAergic interneurons and oligodendrocyte precursor cells in the hippocampus. Nat. Neurosci. 7, 24–32. 10.1038/nn1162 14661022

[B50] Lin S.-C. Huck J. H. J. Roberts J. D. B. Macklin W. B. Somogyi P. Bergles D. E. (2005). Climbing fiber innervation of NG2-expressing glia in the mammalian cerebellum. Neuron 46, 773–785. 10.1016/j.neuron.2005.04.025 15924863

[B51] Liu Q. Y. Schaffner A. E. Chang Y. H. Vaszil K. Barker J. L. (1997). Astrocytes regulate amino acid receptor current densities in embryonic rat hippocampal neurons. J. Neurobiol. 33, 848–864. 10.1002/(sici)1097-4695(19971120)33:6<848::aid-neu11>3.0.co;2-0 9369156

[B52] Liu Q. Y. Schaffner A. E. Li Y. X. Dunlap V. Barker J. L. (1996). Upregulation of GABA_A_ current by astrocytes in cultured embryonic rat hippocampal neurons. J. Neurosci. 16, 2912–2923. 8622122 10.1523/JNEUROSCI.16-09-02912.1996 PMC6579057

[B53] Muthukumar A. K. Stork T. Freeman M. R. (2014). Activity-dependent regulation of astrocyte GAT levels during synaptogenesis. Nat. Neurosci. 17, 1340–1350. 10.1038/nn.3791 25151265 PMC4176984

[B54] Nagler K. Mauch D. H. Pfrieger F. W. (2001). Glia-derived signals induce synapse formation in neurones of the rat central nervous system. J. Physiol. 533, 665–679. 10.1111/j.1469-7793.2001.00665.x 11410625 PMC2278670

[B55] Neniskyte U. Gross C. T. (2017). Errant gardeners: glial-cell-dependent synaptic pruning and neurodevelopmental disorders. Nat. Rev. Neurosci. 18, 658–670. 10.1038/nrn.2017.110 28931944

[B56] Ortinski P. I. Dong J. Mungenast A. Yue C. Takano H. Watson D. J.. (2010). Selective induction of astrocytic gliosis generates deficits in neuronal inhibition. Nat. Neurosci. 13, 584–591. 10.1038/nn.2535 20418874 PMC3225960

[B57] Paolicelli R. C. Bolasco G. Pagani F. Maggi L. Scianni M. Panzanelli P.. (2011). Synaptic pruning by microglia is necessary for normal brain development. Science 333, 1456–1458. 10.1126/science.1202529 21778362

[B58] Parkhurst C. N. Yang G. Ninan I. Savas J. N. Yates J. R. III Lafaille J. J.. (2013). Microglia promote learning-dependent synapse formation through brain-derived neurotrophic factor. Cell 155, 1596–1609. 10.1016/j.cell.2013.11.030 24360280 PMC4033691

[B59] Pascual O. Ben Achour S. Rostaing P. Triller A. Bessis A. (2012). Microglia activation triggers astrocyte-mediated modulation of excitatory neurotransmission. Proc. Natl. Acad. Sci. U S A 109, E197–E205. 10.1073/pnas.1111098109 22167804 PMC3268269

[B60] Penzes P. Cahill M. E. Jones K. A. VanLeeuwen J.-E. Woolfrey K. M. (2011). Dendritic spine pathology in neuropsychiatric disorders. Nat. Neurosci. 14, 285–293. 10.1038/nn.2741 21346746 PMC3530413

[B61] Perea G. Navarrete M. Araque A. (2009). Tripartite synapses: astrocytes process and control synaptic information. Trends Neurosci. 32, 421–431. 10.1016/j.tins.2009.05.001 19615761

[B62] Perea G. Yang A. Boyden E. S. Sur M. (2014). Optogenetic astrocyte activation modulates response selectivity of visual cortex neurons *in vivo*. Nat. Commun. 5:3262. 10.1038/ncomms4262 24500276 PMC4075037

[B63] Pfrieger F. W. Barres B. A. (1996). New views on synapse—glia interactions. Curr. Opin. Neurobiol. 6, 615–621. 10.1016/s0959-4388(96)80093-6 8937825

[B64] Pizzorusso T. Medini P. Berardi N. Chierzi S. Fawcett J. W. Maffei L. (2002). Reactivation of ocular dominance plasticity in the adult visual cortex. Science 298, 1248–1251. 10.1126/science.1072699 12424383

[B65] Pyka M. Wetzel C. Aguado A. Geissler M. Hatt H. Faissner A. (2011). Chondroitin sulfate proteoglycans regulate astrocyte-dependent synaptogenesis and modulate synaptic activity in primary embryonic hippocampal neurons. Eur. J. Neurosci. 33, 2187–2202. 10.1111/j.1460-9568.2011.07690.x 21615557

[B66] Risher W. C. Patel S. Kim I. H. Uezu A. Bhagat S. Wilton D. K.. (2014). Astrocytes refine cortical connectivity at dendritic spines. Elife 3:e04047. 10.7554/eLife.04047 25517933 PMC4286724

[B67] Schafer D. P. Lehrman E. K. Kautzman A. G. Koyama R. Mardinly A. R. Yamasaki R.. (2012). Microglia sculpt postnatal neural circuits in an activity and complement-dependent manner. Neuron 74, 691–705. 10.1016/j.neuron.2012.03.026 22632727 PMC3528177

[B68] Seifert G. Schilling K. Steinhauser C. (2006). Astrocyte dysfunction in neurological disorders: a molecular perspective. Nat. Rev. Neurosci. 7, 194–206. 10.1038/nrn1870 16495941

[B69] Shemer A. Erny D. Jung S. Prinz M. (2015). Microglia plasticity during health and disease: an immunological perspective. Trends Immunol. 36, 614–624. 10.1016/j.it.2015.08.003 26431939

[B70] Shigetomi E. Tong X. Kwan K. Y. Corey D. P. Khakh B. S. (2012). TRPA1 channels regulate astrocyte resting calcium and inhibitory synapse efficacy through GAT-3. Nat. Neurosci. 15, 70–80. 10.1038/nn.3000 22158513 PMC3282183

[B71] Sorg B. A. Berretta S. Blacktop J. M. Fawcett J. W. Kitagawa H. Kwok J. C. F.. (2016). Casting a wide net: role of perineuronal nets in neural plasticity. J. Neurosci. 36, 11459–11468. 10.1523/JNEUROSCI.2351-16.2016 27911749 PMC5125213

[B72] Stevens B. Allen N. J. Vazquez L. E. Howell G. R. Christopherson K. S. Nouri N.. (2007). The classical complement cascade mediates CNS synapse elimination. Cell 131, 1164–1178. 10.1016/j.cell.2007.10.036 18083105

[B73] Stipursky J. Romão L. Tortelli V. Neto V. M. Gomes F. C. A. (2011). Neuron-glia signaling: implications for astrocyte differentiation and synapse formation. Life Sci. 89, 524–531. 10.1016/j.lfs.2011.04.005 21569780

[B74] Tan Z. Liu Y. Xi W. Lou H.-F. Zhu L. Guo Z.. (2017). Glia-derived ATP inversely regulates excitability of pyramidal and CCK-positive neurons. Nat. Commun. 8:13772. 10.1038/ncomms13772 28128211 PMC5290168

[B75] Tremblay M.-È. Stevens B. Sierra A. Wake H. Bessis A. Nimmerjahn A. (2011). The role of microglia in the healthy brain. J. Neurosci. 31, 16064–16069. 10.1523/JNEUROSCI.4158-11.2011 22072657 PMC6633221

[B76] Tsai H.-H. Li H. Fuentealba L. C. Molofsky A. V. Taveira-Marques R. Zhuang H.. (2012). Regional astrocyte allocation regulates CNS synaptogenesis and repair. Science 337, 358–362. 10.1126/science.1222381 22745251 PMC4059181

[B77] Ullian E. M. Harris B. T. Wu A. Chan J. R. Barres B. A. (2004). Schwann cells and astrocytes induce synapse formation by spinal motor neurons in culture. Mol. Cell. Neurosci. 25, 241–251. 10.1016/j.mcn.2003.10.011 15019941

[B78] Ullian E. M. Sapperstein S. K. Christopherson K. S. Barres B. A. (2001). Control of synapse number by glia. Science 291, 657–661. 10.1126/science.291.5504.657 11158678

[B79] Um J. W. Ko J. (2013). LAR-RPTPs: synaptic adhesion molecules that shape synapse development. Trends Cell Biol. 23, 465–475. 10.1016/j.tcb.2013.07.004 23916315

[B80] van Berckel B. N. Bossong M. G. Boellaard R. Kloet R. Schuitemaker A. Caspers E.. (2008). Microglia activation in recent-onset schizophrenia: a quantitative (*R*)-[^11^C]PK11195 positron emission tomography study. Biol. Psychiatry 64, 820–822. 10.1016/j.biopsych.2008.04.025 18534557

[B81] Vitellaro-Zuccarello L. Calvaresi N. De Biasi S. (2003). Expression of GABA transporters, GAT-1 and GAT-3, in the cerebral cortex and thalamus of the rat during postnatal development. Cell Tissue Res. 313, 245–257. 10.1007/s00441-003-0746-9 12898208

[B82] Voineagu I. Wang X. Johnston P. Lowe J. K. Tian Y. Horvath S.. (2011). Transcriptomic analysis of autistic brain reveals convergent molecular pathology. Nature 474, 380–384. 10.1038/nature10110 21614001 PMC3607626

[B83] Volterra A. Meldolesi J. (2005). Astrocytes, from brain glue to communication elements: the revolution continues. Nat. Rev. Neurosci. 6, 626–640. 10.1038/nrn1722 16025096

[B84] Wu Y. Dissing-Olesen L. MacVicar B. A. Stevens B. (2015). Microglia: dynamic mediators of synapse development and plasticity. Trends Immunol. 36, 605–613. 10.1016/j.it.2015.08.008 26431938 PMC4841266

[B85] Xia M. Zhu S. Shevelkin A. Ross C. A. Pletnikov M. (2016). DISC1, astrocytes and neuronal maturation: a possible mechanistic link with implications for mental disorders. J. Neurochem. 138, 518–524. 10.1111/jnc.13663 27187935

[B86] Yang J. Yang H. Liu Y. Li X. Qin L. Lou H.. (2016). Astrocytes contribute to synapse elimination via type 2 inositol 1,4,5-trisphosphate receptor-dependent release of ATP. Elife 5:e15043. 10.7554/eLife.15043 27067238 PMC4829431

[B87] Zhang J.-M. Wang H.-K. Ye C.-Q. Ge W. Chen Y. Jiang Z.-L.. (2003). ATP released by astrocytes mediates glutamatergic activity-dependent heterosynaptic suppression. Neuron 40, 971–982. 10.1016/s0896-6273(03)00717-7 14659095

[B88] Zoghbi H. Y. Bear M. F. (2012). Synaptic dysfunction in neurodevelopmental disorders associated with autism and intellectual disabilities. Cold Spring Harb. Perspect. Biol. 4:a009886. 10.1101/cshperspect.a009886 22258914 PMC3282414

